# Reflections on the ethics of recruiting foreign-trained human resources for health

**DOI:** 10.1186/1478-4491-9-2

**Published:** 2011-01-20

**Authors:** Vivien Runnels, Ronald Labonté, Corinne Packer

**Affiliations:** 1 Globalization and Health Equity Research Unit, Institute of Population Health, University of Ottawa, Canada; 2 Faculty of Graduate and Postdoctoral Studies, University of Ottawa, Canada; 3 Faculty of Medicine, Department of Epidemiology and Community Medicine, University of Ottawa, Canada

## Abstract

**Background:**

Developed countries' gains in health human resources (HHR) from developing countries with significantly lower ratios of health workers have raised questions about the ethics or fairness of recruitment from such countries. By attracting and/or facilitating migration for foreign-trained HHR, notably those from poorer, less well-resourced nations, recruitment practices and policies may be compromising the ability of developing countries to meet the health care needs of their own populations. Little is known, however, about actual recruitment practices. In this study we focus on Canada (a country with a long reliance on internationally trained HHR) and recruiters working for Canadian health authorities.

**Methods:**

We conducted interviews with health human resources recruiters employed by Canadian health authorities to describe their recruitment practices and perspectives and to determine whether and how they reflect ethical considerations.

**Results and discussion:**

We describe the methods that recruiters used to recruit foreign-trained health professionals and the systemic challenges and policies that form the working context for recruiters and recruits. HHR recruiters' reflections on the global flow of health workers from poorer to richer countries mirror much of the content of global-level discourse with regard to HHR recruitment. A predominant market discourse related to shortages of HHR outweighed discussions of human rights and ethical approaches to recruitment policy and action that consider global health impacts.

**Conclusions:**

We suggest that the concept of corporate social responsibility may provide a useful approach at the local organizational level for developing policies on ethical recruitment. Such local policies and subsequent practices may inform public debate on the health equity implications of the HHR flows from poorer to richer countries inherent in the global health worker labour market, which in turn could influence political choices at all government and health system levels.

## Introduction

Canada has a long history of formal policies that have encouraged immigration, and accepts "more immigrants and refugees for permanent settlement in proportion to its population than any other country in the world [1]". For some decades this has included migration of foreign or internationally-trained health professionals, who often fill vacancies in rural and under-resourced regions of the country. Like several other developed countries (and particularly the Anglo-American nations), Canada has come to rely upon internationally-trained health human resources (HHR), particularly doctors and nurses, to meet its labour force needs. Of 260 000 nurses practicing in Canada in 2007, 8% of Registered Nurses (RNs), 2% of Licensed Practical Nurses and 7% of Registered Psychiatric Nurses were educated outside of Canada [2]. For physicians, the proportion of internationally-trained graduates is greater: Of 63 682 doctors practicing in Canada in 2007, 22.4% were internationally-educated [3]. Proportions vary by jurisdiction, with some provinces more reliant on foreign-trained health professionals than others. In Saskatchewan, 49% (733/1644) of physicians were internationally-educated, while in Quebec only 11% (1789/16 782) were educated outside of Canada. While the proportion of foreign-trained family physicians practicing in Canada has declined from 31.9% in 1978 to 24.9% in 2008 and for foreign-trained specialists from 29.7% to 21.5% in 2008, immigration and entry into practice of foreign-trained physicians continues in sizeable numbers [3]. For nurses, between 2003 and 2007, 7.9% (20 319) graduated from an international nursing program. Since 2003, the proportion of internationally educated graduates in the Canadian RN workforce has remained fairly constant at between 7% and 8% [2]. Additionally, the supply of internationally-trained health human resources is an assumed factor considered in Canadian HHR modelling and planning initiatives [4].

Along with other high-income destination countries, Canada's gain in HHR from countries with comparatively low densities of health workers has raised questions of fairness and health equity [5]. Draining the HHR of developing countries and compounding the difficulties of delivering health care within them leads to a form of "perverse subsidy" from poorer to richer nations [6-8]. Below certain densities of health care workers, effective coverage of "essential interventions" is not likely [[6], p. 18]. A suggested staffing guideline for health care provision is the Joint Learning Initiative's 2.5 providers/1000 population. When related to two specific interventions, measles immunization and skilled attendance at birth, this ratio is suggested as "a threshold of worker density" [[9], p. 23]. Malawi, at the extreme lower end of the HHR staffing-ratio continuum and below the threshold, has 0.05 doctors/1000 people [9]. In Canada, one of the better served countries, the doctor-population ratio is estimated at 19.2 doctors/1000 people [10].

This situation of staffing inequalities and health inequities raises a number of questions about the ethics of Canadian recruitment practices and policies. In the past decade the negative impact of health worker migration from poorer countries facing high burdens of disease has renewed longstanding debates between 'source' and 'destination' countries over the economics and ethics of any form of recruitment that enables such migration. Destination countries' normative commitments to the Millennium Development Goals (MDGs), for example, are at odds with recruitment or migration policies that enable a flow of employed or employable health workers from poorer nations which are at risk of failing to meet the health targets of these goals. Similarly, the participation of countries such as Canada in the 'International Health Partnership + Related Initiatives', a new (September 2007) multilateral project to operationalize the Paris Declaration on Aid Effectiveness with a focus on the health MDGs, is compromised through the loss of health workers to Canada and other developed countries from those health aid-recipient nations targeted by this Initiative. There are ethical dimensions to the economics of such flows, and complex human rights considerations, consequences and responsibilities which extend beyond those of individual health workers' seeking to migrate [11-13].

In a follow-up study to earlier research on HHR migration from sub-Saharan Africa to Canada [14], we set out to learn from Canadian recruiters working with public health authorities (1) how they conducted their work, and (2) how they viewed the ethics of recruitment of foreign-trained health professionals. In this article we report our methods and an analysis of the findings of semi-structured interviews with HHR recruiters. Quotes from the study's respondents are also used to illustrate some points in the discussion of global policy options. We conclude our discussion by proposing a potential approach to policy development with regard to ethical recruitment practice at the organizational level.

## Methods

After receiving approval from the University of Ottawa Research Ethics Board and the approval of recruiters' employers, we sampled and interviewed recruiters from urban, underserved, rural and northern areas in five Canadian provinces (Ontario, Manitoba, Saskatchewan, Alberta, and British Columbia), known to be recipients of health professionals from developing countries, including the sub-Saharan African region (the focus of our previous study). In addition to an introductory letter and consent forms, participants received a copy of the questions to be covered during the interview. (See additional file 1: List of interview questions for regional health authorities and hospitals). We conducted interviews with 26 persons responsible for recruiting doctors, nurses and allied health professionals for publicly-funded acute health care organizations. We did not interview recruiters associated with province-wide initiatives, or hospitals that served psychiatric, geriatric, developmentally handicapped and rehabilitation populations or with fewer than five permanent beds, or private hospitals that are funded outside public health plans. All interviews were digitally recorded and transcribed. For the analysis of the interviews, we used qualitative description as an approach [15]. We organized data in response to the questions that we asked. For example, in response to "What types of advertising or recruitment strategies does your organization generally employ?" we included all types of advertising and recruitment strategies. Subsequently, we reviewed the data iteratively for common themes so that, beyond direct description, our analysis was grounded in the data [16,17]. We also paid attention to the language that participants used with regard to issues of an ethical nature. Our analysis was therefore informed by our analytic method, our previous research [8,14], and the literature. The findings are reported here using direct quotes from the respondents.

## Results

### Recruiting foreign-trained health professionals

Recruiters are the end-users of organizational and governmental policies with respect to HHR planning. They do the ground work, interact directly with potential employees and have direct experience of HHR recruitment. The participants in our study were responsible for recruiting different health professionals (nurses, doctors and allied health professionals) while some were responsible for specifically recruiting nurses and/or doctors.

Respondents stated that they did not directly recruit foreign-trained health professionals either outside or inside of Canada, with the exception of two health organizations that were actively recruiting internationally. 'Directly' with respect to international recruitment meant specific, sometimes personally specific, targeting of foreign-trained health professionals in their countries of origin. International recruitment, for most respondents "is not a strategic thrust for us at all," that "we don't go knocking on anybody's door outside of North America." Another recruiter noted, "I shudder at the word 'recruit' internationally because... other than offering information, we're not actively soliciting them." As another reported, "we are not ... trying to lure physicians from their home country." Most respondents reported their organizations neither recruited nor employed many internationally-trained health workers: their representation in the workforce was typically estimated to be less than 5% of their total health organization workforce. This figure is substantially lower than the hard data on the number of foreign-trained doctors and nurses working in the Canadian health care system, but could be a result of our focus on HHR employed by public health authorities and not those working in publicly-financed but privately-run practices. Anecdotally we heard of small towns actively recruiting physicians to establish such practices, but this form of recruitment was not part of our study.

Some recruiters referred to health organizations known to them that either conducted HHR recruitment efforts overseas or authorized third parties to conduct efforts on their behalf. Independent of the interviews, the authors also found evidence in the popular press of health organizations conducting international recruitment trips. The trip(s) to the Philippines by a Saskatchewan health authority in 2008 to recruit nurses is one example [18].

### Recruitment and international advertising

In order to reach potential recruits, recruiters placed advertisements on their organizational websites. Some recruiters also used internet classified advertising such as Workopolis, Monster.ca, and Charity Village. Because the internet is internationally accessible, any internet-based advertising or recruitment campaign implied international reach. As one recruiter said, "Whether you're in South Africa, in India or in Regina, Saskatchewan or in Ottawa... the information is the same, the message is the same, the opportunity is the same." Internet advertising did not always assist the recruitment effort. Recruiters suggested that most applications received through their own and classified websites from foreign-trained candidates were unsuitable, because candidates' qualifications and experience did not match or meet the desired or expected quality that recruiters were seeking.

No-one interviewed indicated they recruited directly through advertisements in foreign academic medical journals, "we're not sending ads to South Africa." This was in keeping with informants' statements of not targeting internationally-trained HHR, and also aligned with a general feeling that printed materials were not a particularly effective means of recruiting candidates. While many academic journals are available electronically through the internet, advertisements in these journals were often only available in hard print format. Nonetheless, the authors found evidence of direct advertisements from Canadian health organizations posted in the printed versions of the South African Medical Journal. Issue 9 of Volume 97 of the printed South African Medical Journal, for example, features some of these advertisements [19]. These advertisements suggest that some parties other than the recruiters we interviewed believed in the chance of successful hires through printed journal advertisements, and did target countries with known HHR shortages.

### Views of third party recruitment

For purposes of our study, third parties are 'for-profit' organizations and agencies that provide contract services to health organizations. Third party recruiters may perform roles that health authority recruiters and human resources staff do not perform, such as recruit directly in developing countries. They may be based or have branch offices in other countries than Canada. For some organizations, third party recruiters or 'head hunters' have provided a 'last chance' method for recruitment. Study participants were generally resistant to using third party recruiters and explained this resistance as related to costs: "I would say within the last five years we have probably only gone and used a head hunter, my recollection is probably twice...and ...it's expensive... it's a lot of patient care money that we'd have to divert."

### The enabling role of recruiters

Before and on arrival in Canada, foreign-trained health professionals need to take certain steps with regard to immigration, education, regulatory and licensing processes before employment can be obtained. System requirements present a number of challenges to foreign-trained health professionals. Licensing, regulation, and education, for example, operate independently and separately from immigration and settlement processes, and at different levels of government. Recruiters reported that these processes, which are not currently coordinated, are lengthy, may present obstacles and delays, and can incur substantial personal costs to the potential HHR worker before he or she can gain employment.

Recruiters felt that foreign-trained health professionals were rarely fully informed as to the processes and the time required to gain a license to practice in Canada in conjunction with all other necessary steps to work in Canada. Recruiters have current knowledge of what foreign-trained HHR need to navigate the multiple systems successfully. They took on supporting roles of helping health workers negotiate these complex systems requirements, even though their own health organization may not ultimately receive the enquiring foreign-trained health professional as an employee. "We offer them information and we'll help them along their path and some day if they're ever licensed [and] eligible and ... want to work here we'll help them with that too." Recruiters also reported that 'the richer' provinces were more attractive to potential employees, not only for reasons of better employment and remuneration packages, but also because regulatory processes in these provinces were easier and faster for applicants to negotiate. (In Canada, most publicly provided health services and professional regulation fall under the mandate of its ten provinces and three territories).

Respondents did not speak specifically about the much-touted urban examples of a taxi driver or pizza delivery man who was a doctor in his home country before coming to Canada. However, there were stories of male and female physicians who abandoned medicine as a result of system challenges. As this recruiter expressed, "there's kind of a mixed bag ... some made it, others didn't. Those are the folks that you'll hear about driving cabs because they were marooned...And believe me I've dealt with a few of those. Some of them gave it up, went and sought other occupations, others luckily went to the States on the IMG (International Medical Graduate) programs... they were a little bit more welcoming." Another told a story of "an experienced specialist ... who needed a residency which (the specialist) didn't get. They ended up putting their funds into a corner store, and the doctor never did go back to medicine."

Systemic challenges are not confined to foreign-born and foreign-trained health professionals. Similar delays and setbacks in the education and licensing processes applied to Canadians who received their medical training overseas, and who were not given preferential treatment on their return, sometimes becoming "lost" to Canada: "They don't realize what's going to happen, they go overseas for their training and expect to come back and (think that) it's going to be quite easy". Whilst recognizing the problems with the "long and cumbersome" process, recruiters appreciated that licensing and regulatory systems are directly connected with quality assurance and public protection. One recruiter compared this to situations where money, inappropriately offered and accepted, may pave a way to qualifications and credentials. "It's a good system because it's very difficult for ... somebody who has money to compromise the system."

### Policies on ethical recruitment

Recruiters reported "a commitment to excellence" in their work, and the conduct of recruitment in professional, considerate, respectful and exemplary ways. Staff physician recruiters, for example, have set up a professional association, the Canadian Association of Staff Physician Recruiters (CASPR), and members are guided by a code of practice. However, recruiters' work was conducted in environments where there were few or no policy guidelines for their work. Only a small number of recruiters referred to organizational policy or any ethical guidance on recruitment of foreign-trained health professionals from either their organization or regional authorities. The issue was not thought to be a high policy priority matter for organizational boards.

We found some ethical statements at the organizational level that were publicly available. The Saskatoon Health Region Nurse Recruitment Trip to the Philippines, mentioned earlier, has an ethical statement, which features 'terms of reference' for organizations directly recruiting internationally-trained HHR in the Philippines [20]. The same region has a statement which expresses the organization's ethics policy called *Our Values in Action *[21]. Despite such examples, it is difficult to avoid concluding that there is little health organization policy in Canada on ethical recruitment of internationally-trained HHR. Those organizations which do have policy still risk depleting overseas hospitals of experienced nursing staff. Again in the case of the Saskatoon nurse recruitment, while its policy restricted hospital nurse recruitment to not more than 5 experienced staff per hospital department, recruitment would lead the Philippine hospitals to fill those now vacant positions with lesser or newly qualified staff [22]. While the politics of nurse migration from the Philippines are complex (the government has an official policy of exporting labour for foreign currency remittances and the health system is highly privatized with insufficient positions for the number of nurses trained), the deliberate export policy has seen a greater than 50% decline in the nurse/patient ratio in the country's public (provincial and district) hospitals, from one nurse per 15-20 patients in the 1990s to one nurse to between 40-60 patients [23].

### Discourses on health worker migration and policy responses

The Saskatoon case brings to light some of the complex ethical and policy issues involved in recruitment or management of global HHR flows. Our interviews explored this topic by asking participants to comment on a range of policies which have been proposed in the literature or other studies to prevent HHR migration from compromising access to health care in under-resourced developing countries.

The policy discussions brought forward connected and intersecting themes which we characterized as two major discourses: a market-based discourse that focused on market responses to labour shortage, and an ethical discourse that included discussion of human rights and matters of 'legality', and 'criminality'.

#### 1) Market-based discourse: shortages, competition and planning failures

The dominant view expressed by participants was that the need to recruit foreign-trained HHR resulted from shortages closely linked to planning failures. The recruitment of internationally-trained HHR was an integral part of solutions to shortage: "(This is) a viable and potentially needed tactic to ensure our community of the services they need." Others saw foreign-trained health professionals as a last resort for filling vacancies because of the effort and resources that were required to bring them on board: "Recruitment starts at home...when you look at a pie...there's four pieces and three of them are local or national solutions. Only one is international... going for an international solution is always your last resort in terms of effort." Echoing this sentiment, and expressing some concern with Canada's reliance on foreign-trained HHR, another recruiter commented "we need to do a whole lot more here...we need to be doing things on our own rather than going and taking from the other countries."

Because of the shortage, recruiters were engaged in a competitive labour market for both domestically and internationally-trained HHR: for the most part this was in the context of interprovincial competition. Recruiters did not specifically refer to the idea of a global labour market in HHR, although recruiters were aware of the global mobility of health professionals, not only for work but also for training and education. Canada was mostly viewed as an end-point of migration, with the possible exception of the United States. There was some speculation of future return to some countries of origin: "we're hearing that ... the Chinas and Indias of the world are ... starting to lure back some of their expatriate professionals...they have tremendous amounts of work and they need them back." At present, however, returning numbers of Indian physicians are a small percentage of the number who seek residency placements in the USA each year, questioning whether such a 'reverse flow' is of sufficient magnitude to overcome initial losses [24].

The need to recruit internationally-trained health professionals was also related to a failure of Canadian HHR planning to meet its labour market requirements: "We wouldn't need to do international nurse recruitment if we had enough resources in our own country, and to grow your own strategy is always an ideal strategy." Responsibility for planning was firmly placed in the hands of governments. Some attributed the problem of shortage to government decisions made several years ago: "All of these different types of recruitment initiatives such as going after foreign trainees (are) done to offset poorer planning. If we hadn't cut the number of health care seats in the early '90s we'd probably be in a different situation right now." Another recruiter stated, "governments decided we had too many doctors in Canada...There was an example of planning stupidly done and stupidly executed." These comments referred to the consequences of steps taken in response to an influential report published in 1991 which examined physician resource management in Canada from the perspective of oversupply of physicians [25].

#### 2) Ethical discourse: professional conduct and international responsibility

Participants took ethics to mean different things, often blurring their responses to include ethics committees at hospitals dealing primarily with research studies. But several respondents also associated ethics with recruitment practice and behaving in a professional manner. One recruiter compared the professional behaviour of a recruiter with a third party recruiter:

They have more latitude than we do in terms of going out and actively searching for the person. (Interviewer: What do you mean by 'latitude'?) Well they can contact somebody in another organization. (Interviewer: - Whereas you would have restrictions on doing that?) Yeah, it's kind of unprofessional for me to be doing that whereas the head hunter will phone and start the conversation, 'Do you have any ideas? Do you have anyone who you know?' So, it's more of an open discussion than if I approach someone...That's somewhat frowned upon.

Ethics was also equated with responsibility and awareness of the implications of recruiting from developing nations. Although participation in our study led some recruiters to think about ethical aspects of internationally-trained HHR recruitment for the first time, there was also some awareness of "responsibility outside of our national issues" and "international responsibility to ensure that developing countries' infrastructure, including their people working in health care, ...is respected and not irrefutably damaged by us recruiting the professionals from their country."

Recruiters' cognisance that recruitment may do harm to source countries was accompanied by a strong suggestion of a dilemma that recruiters faced: "... those countries need these health professionals, and then there's the other side with us trying to recruit to meet our own need... you have those ethical concerns." Another said:

I was watching a documentary about South Africa and felt absolutely horrible. It was a nurse practitioner who ran a clinic ... people had to walk for a day and a half with a sick child just to see the nurse and there were no physicians...she was begging countries like Canada and the US not to take physicians. So it was a little heart wrenching... we need the physicians and the physicians want to get out of those countries. Yet, there were so many people that needed their services you feel a little guilty doing it so you've got mixed emotions about the whole thing.

Another recruiter made a distinction between active and passive recruitment of foreign-trained HHR:

The definition of ethical codes would be a key debate ...For instance, the third party consultant says 'I have people who really want to leave South Africa and they're going to leave, whether they come to you or go to somebody else.' That's a different ethical question than me going in to a hospital in South Africa and walking up to five physicians and saying I want to take you out of this and I want to take you back to (my province). To me there's an ethical difference there. I wouldn't be supportive of the example that I've just used for walking in and just plucking people out but the ethics is different if somebody is going to leave anyway.

Active and targeted recruitment is 'discouraged' in existing codes of ethics [[26,27], p. 4]. But making a distinction between active and passive recruitment is clearly more complex than the direct encounter of a recruiter with a potential employee [8,14,28]. Moreover, the argument that third party recruitment poses different ethical concerns than direct recruitment does not stand up to scrutiny: the net outcome is the same, and procedurally all that differs is the presence of an intermediary. The same argument applies to the earlier statement of recruiting only from North America. If any of the HHR recruiters in North America actively sought health workers from developing countries (whether directly or via a third party agency) a successful North American recruitment leaves a vacancy somewhere that is likely to be filled by such a health worker.

For the most part, recruiters made no claims to any great knowledge of the international situation with regard to HHR, "we don't pretend to be experts by any means in international situations and politics." Recruiters' ethical focus was on doing their jobs professionally and satisfactorily enough to meet personal performance expectations and the needs of their employers.

Recruiters did not perceive themselves as wholly constrained from passively or actively recruiting and hiring foreign-trained health professionals despite personal ethical conflict. Regardless, it was noted that respondents' discussion was peppered with language associated with theft, reflecting an ethical dilemma. For example, "Are we robbing one country to kind of save another?" and "If you're robbing Peter to pay Paul, it's not a sustainable tactic." And "...should we be robbing the other countries who are already short (of HHR)?" The idea of "robbing", however, was discounted and justified by reference to labour market conditions in source countries: "We targeted our efforts (where) ...there are literally thousands of unemployed or underemployed health care professionals", and, "In some countries there's actually an abundance of nurses...Or there's no funding. So, you know, is there an ethical issue? If they're unemployed...I don't think there is."

Other recruiters recognized that people were leaving source countries for reasons other than unemployment, because of "the political situation, the fear for safety." A reference to source countries which have used bonding or other similar requirements to retain health workers was also seen as wrong: "...it's so bad they want to get out of there and if their country is forcing them to stay - that's not good for the individual." The literature also suggests that the migration of health professionals is highly associated with untenable working and living conditions, poor rates of remuneration or lack of professional advancement [14,29,30].

Finally, recruiters strongly supported the individual's right to migrate: "it's the right of those individuals to go where they (want to) go...you know, free migration is free migration."

## Discussion

These two discursive themes - market forces and ethical considerations - reflect those common in global debates surrounding policy and HHR recruitment. Recruitment is basically a response to address market shortages and dominated our participants' responses: "I think it'll be a policy of more how we recruit, not a policy on whether we should or not. Because in all honesty if someone meets the criteria to be licensed...and meets all the...criteria for practice then where their country of origin is, is not necessarily of any consequence." Ethical considerations, notably those of restitution to under-resourced source countries for their human capital losses, remained secondary. Yet by extension, a market conceptualization of labour supply and demand commodifies HHR, and implies some form of repayment to sending countries for foregone training investments or other economic or general welfare losses, quite apart from those nominally offset by private remittances. Such transfers could be affected through bilateral aid or other financial assistance, or by forwarding to the source country for a period of time a portion of income taxes paid by emigrating health professionals working as such in their destination countries.

While some questions have been raised around the methods and models of measuring and forecasting shortages of HHR [31], a recent Canadian report suggests that there is a need to understand better the impacts that health professionals have on health systems and outcomes. Italy, for example, has twice as many doctors per capita as Canada, yet it has no significant differences in life expectancy [32]. Assessing the mix of health workers involved in providing care and changing the way in which care is organized may result in improved efficiency without necessarily increasing physician or nurse supply [32]. Others argue that reduction in the size of the workforce as part of reform may not necessarily lead to efficiencies: shortages of workers make it difficult to achieve organizational reforms or to introduce new technologies [33,34].

Respondents in our study thought that an emphasis on training lesser-skilled health workers who could perform certain tasks in lieu of doctors and nurses in areas of shortage was acceptable. This view also supports a renewed interest in the training of community health workers in southern African countries [35,36]. But another rationale sometimes offered for such training - that it makes such workers less attractive for migration to developed countries - was considered unacceptable: "It's saying basically, take people that are less skilled, don't train them as well so we don't steal them." And another recruiter said "Well it doesn't feel right to me because there are patients at the other end of the care line, right? And so your patients drive what level of care and what level of skilled health care worker you need and so it sounds like you'd train lesser skilled people just to keep them in their home country, and that doesn't really sound very ethical to me." Another respondent said "I think that's just ridiculous. Why should their options be limited? Why shouldn't they be all they can be where they are? I think that's an abhorrent idea. It's terrible." The tenor of such responses surprised us. While it could reflect, in part, the extent to which the two dominant health professions (medicine and nursing) have claimed monopoly rights over practice internationally, it could also represent recruiters' moral concern over all persons having access to the best care possible. But it leaves unaddressed two known facts: that many health problems do not require the level of training that goes into producing physicians and nurses, making expansion of alternative categories of health workers attractive to under-resourced poorer countries; and that, by reducing the chance of employability of such health workers in wealthier countries, it does reduce the economic incentives to migrate.

Although our interviews probed respondents on human rights arguments surrounding the recruitment of HHR, these were not a primary focus by the study's respondents. Nonetheless, the international human rights framework does constitute a critical normative and ethical discourse on HHR migration, albeit one somewhat constrained by competition between individual and collective rights within different human rights treaties [37]. The right of health workers to migrate, for example, may compete with the right of other individuals to have access to core health services [11]. Some human rights scholars argue for a hierarchy of rights, placing some as more basic than others (such as the right to health) and underscoring the principle that all human rights should give disproportionate emphasis to more vulnerable populations (thereby emphasizing the impact of health worker migration on poorer source countries) [38]. Others, including the former UN Special Rapporteur on the Right to Health, contend that Article 12 of the ICESCR obliges some form of financial restitution by highly resourced destination countries to poorly-resourced source countries [39]. Lack of global enforceability, however, makes it unlikely that recourse to the international human rights framework will resolve issues of global health inequities arising from international HHR migration, at least in any near term. Even breaches of human civil or political rights at the individual recruit level are difficult to demonstrate without complaints being brought forward. There have been reports of qualified nurses who were employed as lower level domestic care workers in private facilities in the United Kingdom, contrary to their expectations [27], and complaints with regard to discrimination on the basis of place of origin were found against the British Columbia (BC) College of Physicians and Surgeons [40]. However, as the author of this latter report noted, "BC does not have the time, and foreign-trained immigrants do not have the resources, to engage in human rights complaints against these bodies on a case by case and organization by organization basis" [[40], p. 17].

Human rights violations such as when one country's actions prevent another country's ability to meet its obligations under Article 12 of the ICESCR are more problematic. They have also given rise to inflammatory legalistic arguments featuring the use of words that suggest that there has been a breach in the law or that crimes have taken place. For example, Singh et al., and Attaran and Walker use the term "poaching" [41,42]. In the Canadian HR Reporter, Butler uses the words "accessories to theft," "receivers of stolen goods" as well as "poaching" [43]. "Raiding" is used by Dauphinée [44]. Through even asking the question "Should active recruitment of health workers from sub-Saharan Africa be viewed as a crime?" Mills implies that these are criminal acts [45]. These authors' works also suggest that distinguishing unethical behaviour from criminal behaviour with respect to the recruitment of HHR from developing countries is a grey area.

It is not seriously contested that HHR recruitment leads to health inequities for some countries although Clemens [46] has argued that the source of the problem lies in the 'push' out rather than the 'pull' in and that a focus on recruitment (or any policy that would lessen the migratory flows without addressing the 'push' or the non-medical sources of high disease burdens in poorer countries) is wrong-headed by attacking the symptom rather than the cause. This underscores a general lack of consensus on the choice of policy and allocation of responsibility for addressing health inequities associated with HHR losses (See, for example, [47]). This quote by a recruiter summarizes what has been termed the 'weakest link' argument in global health. "... if we go and take a developing nation's ... [HHR] ... and we pull them down... We're not making the health care any better for anybody.... All it does is shift the problem. Problems have a tendency to also shift back as well unless you rectify the problem." The 'weakest link' argument holds that untreated pandemics in poorer countries (partly arising from lack of HHR) pose direct risks to other nations: witness the present concern in many developed countries with the spread of pandemic influenza or multiple drug-resistant strains of tuberculosis or HIV/AIDS. Unchecked disease can lead to economic decline in poorer nations and to national and regional conflicts with costs to countries like Canada of UN-sanctioned peace-keeping efforts or increased development assistance transfers. In other words, the health of people in disparate countries is becoming increasingly interlinked. This utilitarianism, quite apart from any other ethical argumentation, partly motivates efforts to establish various codes of practice for HHR recruitment [48] aimed at ensuring, at minimum, mutual benefits between source and receiving countries. But, while the UK's Commonwealth Code of Practice (which remained until the adoption of the WHO Global Code of Practice on the International Recruitment of Health Personnel, the key referent in discussions about 'managed HHR migration,') encourages "the establishment of a framework of responsibilities between governments - and the agencies accountable to them - and the recruits" [[27], p. 5], it is less specific about what such a framework might mean for HHR recruiters and their organizations at the local level.

Given the general absence of organizational policy on recruitment of foreign-trained health professionals in Canadian settings, and the pervasiveness of a market defence of passive recruitment, we believe it is important that a morally defensible recruitment policy be developed. Such a policy would still need to work within the presence of a market dominated environment: that is, while human rights or moral arguments may be important within any policy framing, there is a global labour market dynamic that well-intended statements alone are unlikely to alter.

A corporate social responsibility policy could afford one such approach. Corporate social responsibility (CSR) has been defined as "a configuration of principles of social responsibility, processes of social responsiveness, and policies, programs, and observable outcomes *as they relate to the firm's societal relationships *(author's italics)" [[49], p. 693]. CSR is controversial, its theoretical roots having been described as "complex and unclear" [[50], p. 51], and its practices remaining "prey to the vagaries of the market" [51] - meaning CSR is disposable when it collides with corporate bottom lines. More pointedly, corporate social responsibility has been critiqued as a way of branding corporate 'goodness' and the use of company developed 'self-regulation' a diversion to "avoid mandatory regulation or to defuse public pressure" [52]. Applied to public bodies such as hospitals and regional health authorities that are publicly accountable and not driven by profit-margins, however, the basic tenets of corporate social responsibility could assist such organizations in grappling more credibly with the domestic and global equity implications of HHR migration [53]. Such tenets would include, at a minimum:

• Public disclosure of a health organization's policy and practices with respect to recruitment of foreign-trained HHR

• Monitoring of its recruitment practices

• Public recognition of domestic and global health equity implications of the global flow of HHR, notably from poorer, under-resourced to wealthier and (comparatively) better- or even over-resourced countries

• Statements on how the organization might seek to mitigate the health inequities of such flows - regardless of whether there is active or passive recruitment

• Requirements for recruitment practices of third party agents it might employ in filling its HHR needs

Health organizations can adopt their own policies to help ensure a locally and ethically defensible approach to the recruitment of foreign-trained health human resources. Local health organizations may not be mandated to engage in, for example, increased health development assistance to poorer source countries (which has been shown to reduce the rate of outward health worker migration) or the type of bilateral tax agreements mentioned earlier by which a portion of émigré health workers' taxes are transferred to the health or education systems of the countries they left [54]. But they are certainly free to advocate, individually and collectively, for such measures at higher national government levels or to engage in public awareness campaigns urging greater global generosity in supporting growth in the public health systems of poorer countries. In addition, ethical arguments create a moral imperative for intervention of some sort. Notable here is the theory of relational justice [55-57], which holds that the global gap between rich and poor (which undergirds much health worker migration) is an effect of past violent histories and present institutional rules that favour already wealthier nations. This places demands on beneficiary institutions and individuals within them, as moral actors, to engage in some forms of restitution that would, if not eliminate, then at least reduce the scale and scope of the poverty and other globally-affected socioeconomic conditions that create both higher disease burdens and fewer health workers in many of today's HHR source countries.

## Conclusions

The recruiters that we interviewed are conscientious, caring and professional in their efforts to employ and settle foreign-trained recruits. Recruiters personally have little involvement in setting broader HHR policy direction or policy making. An absence of organizational, provincial and national level policies and commitment to international guidelines such as the Commonwealth Code of Practice for the International Recruitment of Health Workers [27] also suggests that recruiters and employing organizations have little in the way of resources to respond to questions of the ethics of recruiting foreign-trained workers that result in sending country inequities. Similarly, the adoption of the WHO Global Code of Practice on the International Recruitment of Health Personnel by the World Health Assembly in 2010, whilst providing an example of the good intentions of participating member states, may exert insufficient influence in deterring some recruitment practices because of the difficulties of implementing the Code in widely disparate organizational settings and the voluntary and unregulated nature of the Code itself.

How individuals are treated ethically by the country that recruits them forms part of a different and larger set of questions that lie outside the professional practice of recruiters. At the country level, current failures to ascribe to a code of practice or to develop policy with regard to recruitment of foreign-trained health professionals reflects a strong propensity to continue with the default discourse - that of the market - and for governments and organizations to deal with ethical responsibilities as "a matter of sublime irrelevance" [53]. As one recruiter put it "The importance of policy is really quite key. I do appreciate it but who has time to sit and develop [it]?" Recent history of global health workforce efforts strongly suggests that recruitment of foreign-trained health professionals from developing countries will continue to be an exercise with little ethical oversight, revisited only voluntarily, and discussed perhaps once in a while when exposed to adverse publicity [42], or when collective consciences are pricked by lobbying and advocacy efforts.

## Competing interests

The authors declare that they have no competing interests.

## Authors' contributions

VR participated in the design of the study, collected data, analyzed data, and drafted and revised the manuscript. RL and CP conceived the study, participated in the design and coordination of the study, helped draft and revise the manuscript. All authors read and approved the final manuscript.

## Supplementary Material

Additional file 1 **Questionnaire**. List of interview questions for regional health authorities and hospitals. Click here for file
