# Supplementary material for: Reflections on the ethics of recruiting foreign-trained human resources for health
Source: Hum Resour Health. 2011 Jan 20;9:2. doi: 10.1186/1478-4491-9-2 (PMC3032656; doi:10.1186/1478-4491-9-2)
Supplement: Additional file 1 — Questionnaire. List of interview questions for regional health authorities and hospitals. [file 1478-4491-9-2-S1.PDF]

Université d'Ottawa · University of Ottawa
Institut de recherche sur la santé des populations
Institute of Population Health

Interview Questions for Regional Health Authorities and Hospitals

Introduction

Thank you for agreeing to participate in this interview. For your reference we have listed the main questions that we will be asking you during the interview. We ask if you would please have a copy of these questions available when we are conducting the interview, particularly for Section 5 (Page 2 and 3) on organization policy where there is a list of options which we would like you to rate. If you have any questions that you would like to pose during the interview, please feel free to do so.

Section 1: Advertising and Recruitment Strategies & Use of Resources

1. What types of advertising or recruitment strategies does your organization generally employ?
2. What types of advertising or recruitment strategies does your organization use for specific positions (as opposed to a general call?)
3. Do you have a website where you advertise your openings?
4. As part of your recruitment efforts do you subscribe to employment websites, such as monster.com, workopolis.com where resumes are posted?
5. Is all of your recruiting and advertising handled internally or do you use a third party for some, or all, of your advertising and recruitment initiatives?
6. Reflecting upon your recruitment and advertising initiatives in general, what is the reach of your initiatives? That is, where do your initiatives usually target? How many countries do your initiatives reach?
7. In addition to formal recruitment and advertising methods, are you aware of informal methods of recruitment? Such as word of mouth, informal networks, or other means?
8. Approximately how many full time equivalent (FTE) staff members does your organization employ for i) recruiting domestically trained health professionals? ii) recruiting foreign-trained health professionals?
9. Do you have an annual budget or any estimates regarding the costs to your organization or region/area for the recruitment of foreign trained health professionals?

Section 2: Foreign Trained Health Professionals

1. Does your organization keep any records of the numbers of foreign-trained health professionals hired and their country of training?
2. Does your organization recruit and/or employ any physicians, nurses or other health professionals from any developing countries?
3. Does your organization recruit and/or employ any physicians, nurses or other health professionals from sub-Saharan Africa (SSA) countries?

1, rue Stewart 1 Stewart Street
Ottawa, Ontario K1N 6N5 Canada Ottawa, Ontario K1N 6N5 Canada
www.iph.uottawa.ca
(613) 562-5660 Télé/Fax (613) 562-5659

4. Would you say that the developing countries, (including sub Saharan countries) which your organization recruits from have changed over time?
5. What countries have you recently started to target or see yourselves targeting in the near future?

Section 3: Immigration Programs and Licensing

1. Has your organization made use of any immigration programs, such as the Provincial Nominee Program, to help settle and or recruit foreign trained health professionals?
2. What do you think about these programs? Have you been satisfied with them? Have there been any problems?
3. Are you aware if the foreign-trained health professionals working in your area or organization are entering on temporary permits or as permanent residents?
4. To your knowledge do foreign-trained health professionals have difficulty obtaining a license to practice in Canada?
5. On average, how long does it take foreign-trained health professionals to become licensed to practice in Canada?
6. Does your organization assist foreign-trained health professionals to obtain their license to practice or to upgrade/advance their skills?

Section 4: Intention to Stay or Return

1. Do you know if most of the foreign-trained health professionals your organization has recruited intend to stay in Canada, or return to their country of origin?
2. To your knowledge, are very few, some, or many health professionals returning to their country of origin?
3. If and when health professionals return, do you know approximately how much time they would have been practicing in Canada?

Section 5: Organization Policy

1. Does your organization have a policy on the recruitment of health professionals?
2. Do people in your organization think that Canada needs to recruit foreign-trained health professionals?
3. Should Canada be looking to recruit more or less foreign-trained health professionals?
4. What would you suggest is the best way to address losses for developing countries whilst balancing individual rights to migrate? Do you have any other suggestions or general thoughts with regard to Health Human Resources migration from developing countries to developed countries?

5. Which of the following policy options might be supported by your organization/department? On a scale of 1-5, where 1 is no support and 5 is high support, how much do you think your organization would support this policy approach?

   a. Ethical codes on recruitment from developing countries where there is an existing shortage of health professionals.
   b. Government reparations to source countries for training and health system loss that has resulted from the migration of health professionals.
   c. Better health human resource planning in receiving countries such as Canada to minimize recruitment outside Canada.
   d. Increased development assistance to strengthen health systems in source countries that minimize some of the health system factors that encourage people to leave, - or 'push' factors.
   e. Requiring mandatory in-country work after graduation of trained health workers in source countries, i.e. for a specified time in a specified location. (sometimes called bonding)
   f. Requiring financial repayment by trained health workers to public training institutes in the case when no in-country work takes place after graduation.
   g. Focus on training auxiliary or lower-skilled workers in source countries, which might decrease their attractiveness to health systems in receiving countries.
   h. Management of migration through bilateral or multilateral agreements that encourage temporary stays only and return health workers to their source country.
   i. Two-way staff flows which allow health workers from the receiving country to provide a period of service to the source country,
   j. Voluntary financial contributions from those who have emigrated to be directed to the health systems of the source country.
   k. Assigning a share of income tax payments made by migrants to a development fund, or some other form of bilateral tax agreement.

6. If you don't think that bilateral or multilateral agreements are feasible or appropriate, do you think that anything else can or should be done?

THANK YOU

1, rue Stewart 1 Stewart Street
Ottawa, Ontario K1N 6N5 Canada Ottawa, Ontario K1N 6N5 Canada
www.iph.uottawa.ca
(613) 562-5660 Télé/Fax (613) 562-5659
